# A Comprehensive Study on the Rejuvenation Efficiency of Compound Rejuvenators for the Characterization of the Bituminous Binder, Mortar, and Mixture

**DOI:** 10.3390/ma15155458

**Published:** 2022-08-08

**Authors:** Mingliang Li, Shisong Ren, Xueyan Liu, Zhe Wu, Haopeng Zhang, Weiyu Fan, Peng Lin, Jian Xu

**Affiliations:** 1 Key Laboratory of Transport Industry of Road Structure and Material, Research Institute of Highway Ministry of Transport, Xitucheng Road No. 8, Beijing 100088, China; 2 Section of Pavement Engineering, Faculty of Civil Engineering & Geosciences, Delft University of Technology, Stevinweg 1, 2628 CN Delft, The Netherlands; 3 Jiangsu Aoxin Science & Technology Co., Ltd., No. 95 Baoying Dadao, Baoying, Yangzhou 225800, China; 4 Sinopec Fuel Oil Shandong Co., Ltd., Tongan Road, Laoshan District, Qingdao 266000, China; 5 State Key Laboratory of Heavy Oil Processing, China University of Petroleum, Qingdao 266580, China

**Keywords:** rejuvenation efficiency, compound rejuvenator, rejuvenated bitumen, asphalt mortar, asphalt mixture

## Abstract

This study aims to comprehensively investigate the rejuvenation efficiency of various self-developed compound rejuvenators on the physical, mechanical, and aging properties of aged bitumen, asphalt mortar, and mixture. The results revealed that the restoration capacity of vacuum distilled-oil rejuvenators on high-and-low temperature performance-grade of aged bitumen is more significant. In contrast, an aromatic-oil based rejuvenator is good at enhancing low-temperature grade and aging resistance. Moreover, the temperature and time of the curing conditions for mixing recycling of asphalt mixture were optimized as 150 °C and 120 min. Furthermore, the sufficient anti-rutting, structural stability, and moisture resistance of recycled asphalt mixture affirmed the rejuvenation efficiency of compound rejuvenators.

## 1. Introduction

Asphalt pavement is one of the essential infrastructures because of its flexibility and versatility characteristics. However, the heavy traffic loading and environmental conditions (oxidation aging and moisture erosion) both limit the service life of asphalt pavement [1,2]. Furthermore, more distresses of asphalt pavement would occur due to poor mechanical performance, such as rutting, cracking, raveling, etc. [3]. Afterward, the maintenance and reconstruction processes are conducted to address the damaged asphalt roads, which leads to lots of reclaimed asphalt pavement (RAP) waste materials being produced. The conventional landfill deposition method of handling these waste materials results in environmental pollution and waste of resources [4]. In addition, the reconstruction of asphalt roads also consumes lots of fresh bitumen and raw aggregates [5,6]. From environmental protection, cost-reduction, and resource-saving viewpoints, the recycling technology of asphalt pavement strongly attracts attention from global pavement-engineering researchers.

It was reported that incorporating RAP materials into asphalt mixtures could reduce the consumption of binder and aggregate and improve the dynamic modulus and rutting resistance [7,8,9]. Nevertheless, the aged bitumen in RAP materials strongly increases the risks of low-temperature and fatigue cracking [10,11,12,13]. Furthermore, the aging reactions of bitumen led to the mutual transformation and proportion imbalance between different chemical components. As a result, the lightweight aromatic fraction was reduced, and the heavy-weight asphaltene molecules increased [14]. Therefore, to balance the distribution of chemical fractions, the petroleum- or bio-based oils with low-viscosity and light-molecular weight were developed as rejuvenators and incorporated into RAP materials to reverse the inferior properties of aged bitumen to virgin binder levels. In general, the rejuvenator has the physical effect of adjusting the viscosity and improving the workability of aged bitumen and chemical/structural functions in the aspects of (1) coordinating the chemical fractions of aged bitumen; (2) enhancing the solubility and dispersing the asphaltene molecules in maltene matrix; and (3) activating the molecular mobility and accelerating the mixing of aged bitumen with virgin binder and aggregates [15].

Different rejuvenators were proposed to renew the properties of aged bitumen, including cooking oil, engine oil, naphthenic oil, aromatic oil, bio-based extraction, etc. [16,17,18,19]. Guo et al. [20] found that the reactivation effect of the warm-mix rejuvenator on the complex modulus of aged binder was more significant than the aromatic oil. Additionally, Suo et al. [21] evaluated the aging behavior of vegetable oil (corn oil and soybean oil) in rejuvenated bitumen and asphalt mixture. They proved that the rheological properties of rejuvenated binders remarkably depend on the rejuvenator type and dosage. Moreover, the vegetable oil rejuvenated bitumen exhibited excellent anti-aging properties, which were associated with the unsaturated carbon chains in vegetable oil. Recently, Bajaj et al. [22] studied the rejuvenation efficiency and mechanism of seven types of rejuvenators with rheological and chemical tools. These rejuvenators were classified into softeners, replenishers, and emulsifiers. The paraffinic oil was only on a physical level, while others (aromatic extracts and bio-oil) interacted with aged bitumen at a chemically level. Regarding the optimum rejuvenator content, Behnood [23] proposed a balance performance design method in which the maximum rejuvenator dosage could be determined to satisfy the high-temperature performance. At the same time, the minimum concentration was related to the restoration degree of both intermediate and low-temperature properties of the asphalt binder and mixture.

However, these commercial rejuvenators show disadvantages regarding high cost, confidential recipe, unstable performance, and poor aging resistance [22]. Developing innovative rejuvenators with cost-effective and anti-aging characteristics manifests important research significance and application prospects. At the same time, comprehensive studies on the rejuvenation efficiency evaluation of rejuvenated bitumen binder, asphalt mortar, and mixture are rarely proclaimed. On the one hand, when only focusing on the rejuvenator optimization for bitumen level, the validation of the rejuvenation efficiency on the asphalt mortar and mixture is lacking [24].

## 2. Research Objective and Methodology

The global objective of this study is to provide a comprehensive evaluation of the rejuvenation efficiency of different self-developed rejuvenators on both artificial- and RAP-aged bitumen, as well as the asphalt mortar and mixture levels. Here are some individual objectives and tasks:

(i) The self-developed compound-modified rejuvenators with different viscosity grades will be manufactured by mixing the virgin bitumen and various pure-rejuvenators with variable rejuvenator-virgin bitumen ratio; (ii) The optimized dosages of self-developed rejuvenators will be calibrated with the penetration-grade standard; (iii) The rejuvenation efficiency of different self-developed rejuvenators on the conventional, rheological, aging, and compatibility properties of artificial- and RAP-aged binders will be evaluated; (iv) The optimum type and dosage of the self-developed compound rejuvenator will be determined for asphalt mortar and mixture levels; (v) The physical and mechanical properties of recycled asphalt mortar and mixture will be characterized further to validate the rejuvenation efficiency of the self-developed compound rejuvenator and accelerate its engineering application.

**Figure 1:**
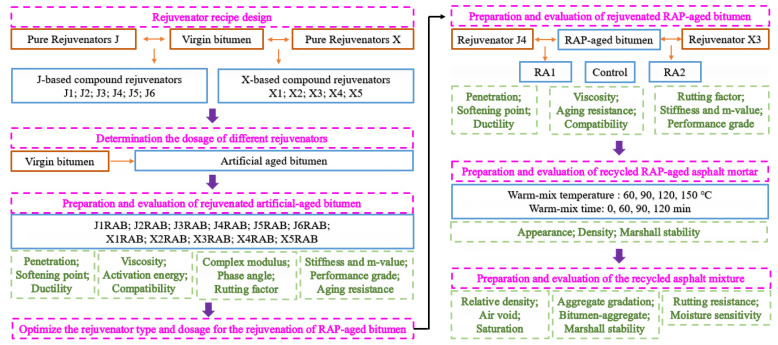
The research protocol of this study.

Figure 1 illustrates the detailed research protocol. Firstly, different compound rejuvenators with various viscosity grades were manufactured by mixing the virgin bitumen and pure rejuvenators at variable mass ratios, including the vacuum distilled oil (rejuvenator J) and aromatic oil (rejuvenator X). Meanwhile, the artificial-aged bitumen was prepared using the air-blowing oxidation method. Then, through restoring the penetration-grade of artificial-aged binder to the virgin bitumen level, the optimum dosages of different compound rejuvenators were determined, and corresponding rejuvenated artificial-aged bitumen was further evaluated in terms of physical, rheological, and aging properties. Afterward, the compound rejuvenator type and dosage were optimized for rejuvenating the RAP-aged binder.

Furthermore, the properties of rejuvenated RAP-aged bitumen were compared with the control sample to verify the rejuvenation efficiency of optimized compound rejuvenators on performance restoration of RAP-aged binder and optimize the rejuvenator type and dosage. Hereafter, the influence of warm-mix temperature (60, 90, 120, and 150 °C) and time (0, 60, 90, and 120 min) on the density and Marshall stability of recycled asphalt mortar was detected first to determine the warm-mix conditions for the regeneration of recycled asphalt mixture using compound rejuvenators. Finally, the recycled asphalt mixtures with the optimum compound rejuvenator type and dosage were manufactured and compared to the fresh ones regarding the volumetric characteristics, rutting, and moisture resistance performance.

## 3. Materials and Characterization Tests

### 3.1. Raw Materials

In this study, the virgin bitumen with the PEN-70 grade was utilized to prepare the artificial-aged bitumen and compound rejuvenators. The physical properties and chemical components of virgin bitumen are listed in Table 1. Moreover, two pure rejuvenators, vacuum distilled oil (rejuvenator J) and aromatic oil (rejuvenator X), were obtained from Jiangsu Province, China. Table 2 displays the conventional properties of two rejuvenators, including the density, viscosity, saturate-aromatic-resin-asphaltene (SARA) fractions, mass loss, and viscosity ratio after the Rolling Thin Film Oven (RTFO) short-term aging procedure [25].

### 3.2. Preparation of Aged Bitumen

**Figure 2:**
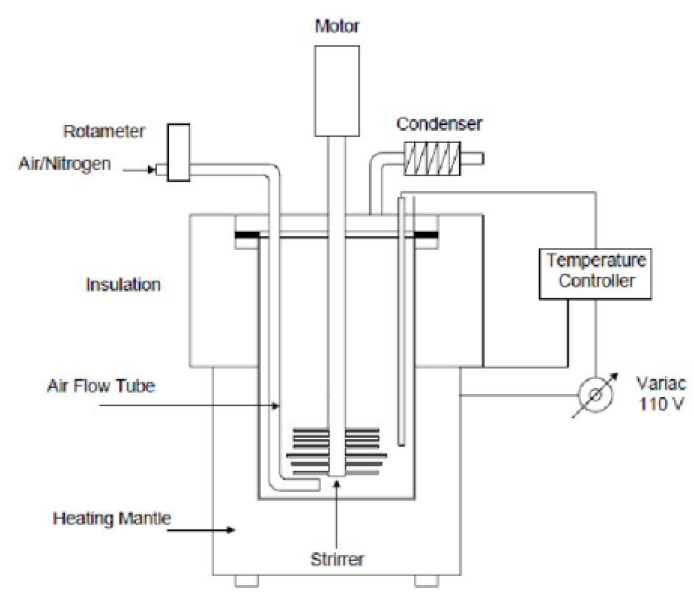
The air-blowing device for oxidation aging of bitumen.

The artificial-aged bitumen was manufactured using a self-developed air-blowing device, shown in Figure 2. The preheated virgin bitumen was dumped into the reaction vessel and heated to 220–230 °C at atmospheric pressure and airflow of 2 L/min [3]. The aging oxidation reaction between the bitumen and pumped oxygen increased the bitumen stiffness. The penetration level of aged bitumen was monitored during the air-blowing aging test. The artificial-aged binder was obtained when its penetration value reached 11 (0.1 mm), close to the aged binder from the reclaimed asphalt pavement (RAP-aged). The basic properties of RAP-aged and artificial-aged binders are presented in Table 3. It was found that their penetration and softening point values are similar, and the artificial-aged bitumen could represent the aged binder in RAP materials to a certain extent.

### 3.3. Preparation of Self-Developed Compound Rejuvenators

In this study, these compound rejuvenators were prepared by mixing the pure rejuvenators (RJ or RX) with virgin bitumen at different rejuvenator-bitumen ratios. Table 4 lists the technical requirements of the hot-mixed asphalt binder [30]. The rejuvenator types were distinguished based on a difference in the 60 °C-viscosity, mass change, and viscosity ratio after short-term aging [31]. With the increase of 60 °C-viscosity, the sequence of six rejuvenator groups was R1, R5, R25, R75, R250, and R500. Moreover, the viscosity ratio of all rejuvenators should be lower than 3.

**Figure 3:**
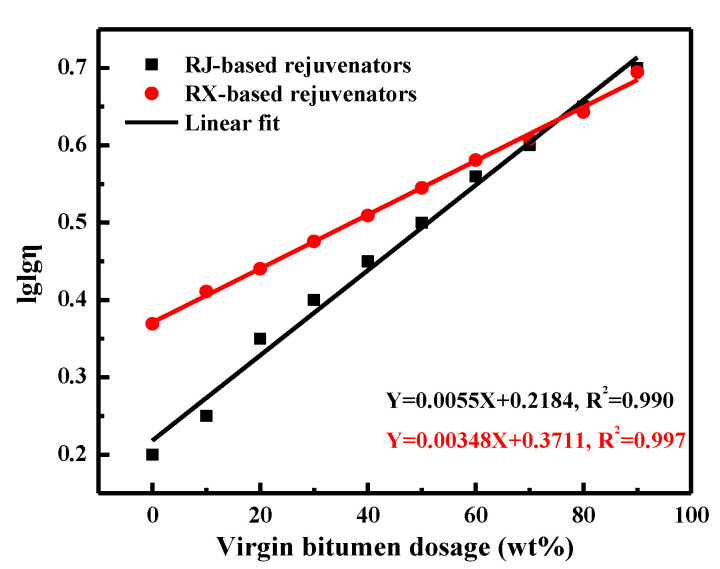
The influence of virgin bitumen dosage on the viscosity of complex rejuvenators.

The virgin bitumen was preheated in an oven at 150 °C and mixed with the pure rejuvenator in an aluminum box for at least 10 min to ensure the homogeneity of rejuvenator-virgin bitumen blends. The 60 °C-viscosity values of compound rejuvenators are illustrated in Figure 3. The compound rejuvenator with a higher dosage of virgin bitumen presents a larger viscosity. In addition, when the virgin bitumen dosage is the same, the viscosity of the RJ-based rejuvenator is lower than that of the RX-based one, and the difference becomes smaller as the virgin bitumen content increases. There is a significant single-logarithm correlation between the viscosity of compound rejuvenator and virgin bitumen dosage:

(1) $$\mathrm{lglg}\left(\eta \right)=k*c+b$$

where η refers to the 60 °C-viscosity, and c represents the content of virgin bitumen in the compound rejuvenator. Compared to the RX-based rejuvenators, the viscosity values of the RJ-based ones are strongly dependent on the virgin bitumen dosage.

To fabricate the compound rejuvenators with different viscosity grades, the mass ratio of the virgin bitumen and rejuvenator RJ is 20:80, 40:60, 60:40, 70:30, 80:20, and 87:13, while, for rejuvenator RX, it is 18:82, 47:53, 60:40, 80:20, and 85:15, respectively. Table 5 displays the viscosity grade, recipe composition, 60 °C-viscosity, and short-term aging properties of different compound rejuvenators. Interestingly, with the increase of virgin bitumen dosage, the viscosity ratio of compound rejuvenators rises gradually, regardless of the pure rejuvenator type. It indicates that the compound rejuvenator with a high content of pure rejuvenator exhibits better aging resistance.

### 3.4. Aggregate Gradation of Asphalt Mixtures

**Figure 4:**
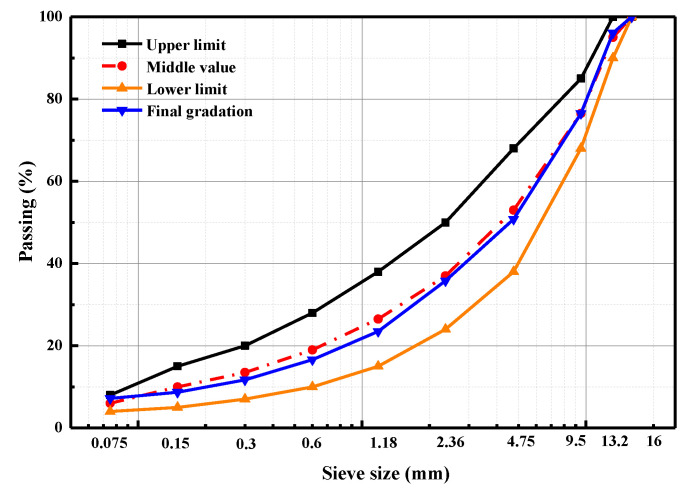
Determination of aggregate gradation for asphalt mixtures.

To further validate the optimum rejuvenator type and mixing condition, the recycled asphalt mixtures were manufactured and evaluated in terms of volumetric characteristics, rutting, and moisture damage resistance. Before the preparation of the recycled asphalt mixture, the aggregate gradation and bitumen–aggregate ratio in RAP material were firstly measured, and the results are shown in Table 6. This study divides the aggregate gradation of RAP materials into three groups: 0–5 mm, 5–10 mm, and 10–15 mm. The control and recycled asphalt mixtures are all AC-13, and the aggregate gradation is shown in Figure 4. The binder in the control asphalt mixture here is the virgin bitumen (AH-70). While preparing the recycled asphalt mixture, the fine aggregates in RAP materials were mixed with rejuvenator X3 for 60 s and cured in an oven at 150 °C for 120 min. Afterward, it was mixed with coarse aggregates and filler at 170 °C for 2 min. It should be mentioned that the aggregate gradation, bitumen–aggregate ratio, and preparation conditions of the control sample are the same as the recycled asphalt mixture.

### 3.5. Characterization Methods

#### 3.5.1. Conventional Tests

The physical properties of the virgin, aged, and rejuvenated bitumen were measured, including the 25 °C-penetration [26], softening point [27], and 15 °C-ductility [28]. Moreover, the shear-viscosity values of binders were tested using the rotational viscometer with the shear rate of 20 rpm at different temperatures of 60, 100, 110, 120, 135, and 150 °C, respectively [32].

#### 3.5.2. Dynamic Shear Rheometer (DSR) Tests

The rheological properties of bitumen were evaluated through DSR tests [33]. The frequency sweep test detected the complex modulus G* and phase angle δ. The loading frequency increased from 0.1 to 100 rad/s at 60 °C. Moreover, the rutting factor G*/sinδ of all bitumen samples was measured with the temperature sweep test. The temperature differed from 58 to 70 °C with an interval point of 2 °C and a frequency level of 10 rad/s [34].

#### 3.5.3. Bending Beam Rheometer (BBR) Test

The BBR method was implemented to estimate the low-temperature cracking resistance of virgin, aged, and rejuvenated binders [35]. The creep stiffness (s) and m-value were measured at −6, −12, and −18 °C [3].

#### 3.5.4. Short-Term and Long-Term Aging Test

To probe the aging behaviors of rejuvenated bitumen, the rolling thin film oven (RTFO) [25] and pressure aging vessel (PAV) [36] tests were conducted to simulate the short-term and long-term aging processes artificially. In the RTFO test, 35 g of bitumen sample was aged in a heated oven at 163 °C for 85 min. Moreover, in the PAV process, the short-term aged bitumen was poured into an airtight container with a high air pressure of 2.1 MPa. The aging temperature and duration were 100 °C and 20 h, respectively.

#### 3.5.5. Performance Characterization Methods of Recycled Asphalt Mortar and Mixture

In this study, the volumetric (density, air void, saturation) and mechanical properties (Marshall stability and dynamic stability) of recycled asphalt mortar and mixture were characterized and compared with the control sample [37]. The wheel tracking test was employed to assess the rutting resistance of the control and recycled asphalt mixture at 60 °C. At the same time, the residue Marshall stability ratio parameter was used to estimate the moisture sensitivity. During the rutting measurement, the asphalt mixture samples were subjected to a repeated wheel load of 0.7 MPa for 1 h [38,39]. The rutting deformation value was recorded constantly, and the dynamic stability DS was calculated as follows:

(2) $$\mathrm{DS}=\frac{\left(t_{2}-t_{1}\right)*N}{\left(d_{2}-d_{1}\right)}$$

where d_1_ and d_2_ refer to the deformation values when the loading time is t_1_ and t_2_, and N is the wheel speed (42 passes per minute).

In addition, two groups of standard Marshall samples of control and recycled asphalt mixtures were fabricated, which were immersed in water for 30 min (dry) and 48 h (wet), respectively [37]. Then, their Marshall stability values of MS0 (dry) and MS1 (wet) were measured. The residue Marshall stability ratio (RMS%) was then calculated as follows:

(3) $$\mathrm{RMS}\%=\frac{{\mathrm{MS}}_{1}}{{\mathrm{MS}}_{0}}*100$$

## 4. Results and Discussion

### 4.1. Determining the Optimum Dosage of Compound Rejuvenator

This study determined the optimum dosages of various compound rejuvenators with diverse viscosity grades according to the penetration-value restoration to the virgin bitumen level [10]. The rejuvenated binders were prepared through mixing the preheated aged bitumen and various compound rejuvenators with different viscosity grades and dosages at 150 °C under a mixing speed of 1500 rpm for 30 min.

**Figure 5:**
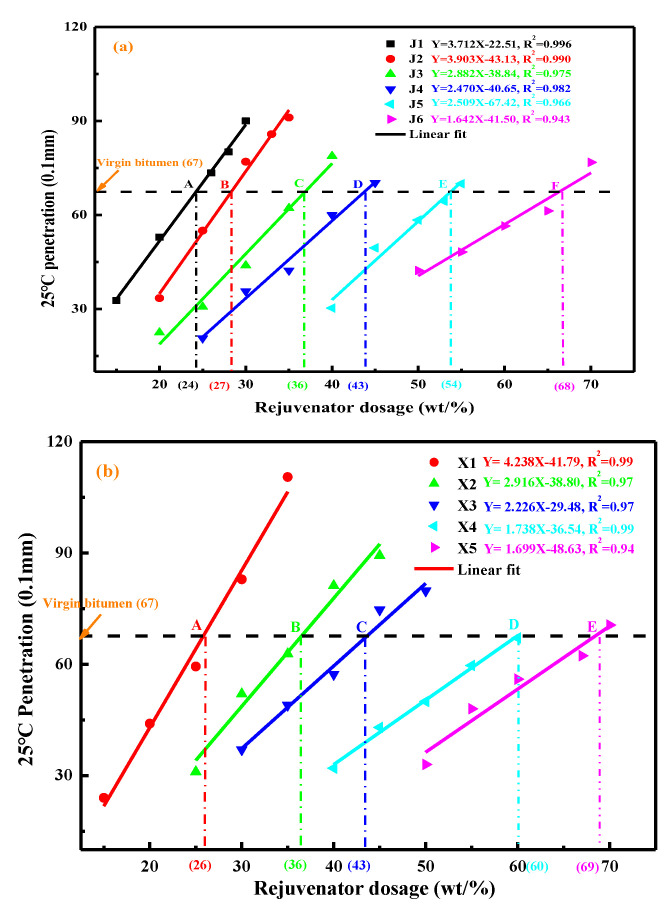
Effects of complex rejuvenator dosage on the penetration of rejuvenated binders. (**a**) RJ-based compound rejuvenators; (**b**) RX-based compound rejuvenators.

The penetration values of rejuvenated bitumen as a function of rejuvenator dosage and type are illustrated in Figure 5. As the rejuvenator content increases, the rejuvenated binder’s penetration-value is recovered gradually. Meanwhile, the compound rejuvenator with higher pure rejuvenator content would rehabilitate the penetration grade of aged bitumen to a greater degree [15]. There is a significant linear relationship between the penetration of rejuvenated bitumen and compound rejuvenator concentration, and the correlation functions are also displayed in Figure 5. With the increment in the virgin bitumen dosage in the compound rejuvenator, the slope value in correlation equations reduces dramatically. It implies that the virgin bitumen exhibits a weaker function in restoring the penetration-grade of aged bitumen than pure rejuvenators [21].

Additionally, the influence of compound rejuvenators on the penetration grade of rejuvenated bitumen also depends on the pure rejuvenator type. When the mass ratio of rejuvenator to virgin bitumen is the same, the rejuvenated bitumen with the RJ exhibits a more considerable penetration value than rejuvenator RX. It is attributed to the softer characteristic of pure rejuvenator RJ, which is consistent with the viscosity result [13]. To restate the penetration-grade of aged bitumen to the virgin bitumen level (6.7 mm), the optimum dosages of compound RJ-based rejuvenators are 24 wt%, 27 wt%, 36 wt%, 43 wt%, 54 wt%, and 68 wt%, respectively. On the other hand, the required contents of compound RX-based rejuvenators are 26 wt%, 36 wt%, 43 wt%, 60 wt%, and 69 wt%, respectively.

### 4.2. Rejuvenation of Artificial-Aged Bitumen

#### 4.2.1. Physical Properties

The physical properties, including the penetration, softening point, ductility, and viscosity, of rejuvenated artificial-aged binders, were measured and compared with virgin bitumen to evaluate the rejuvenation efficiency of different compound rejuvenators with various viscosity grades. Table 7 shows that the physical properties of all rejuvenated binders are close to virgin bitumen, which fulfills the performance requirements of AH-70 bitumen in the JTB F40-2004 standard. It should be mentioned that the acronym of each sample means a rejuvenated artificial-aged bitumen with one compound rejuvenator, while the virgin bitumen is referred to as “VB”.

All compound rejuvenators could restore the physical properties of artificial-aged bitumen to the virgin binder level. The rejuvenated bitumen with a smaller 60 °C-viscosity presents a higher ductility and lower softening point. Overall, although the penetration grades are the same, the high-and-low temperature properties and workability of various rejuvenated artificial-aged binders are significantly different, especially regarding the ductility and viscosity properties.

In addition, the J6RAB rejuvenated bitumen presents the lowest softening point and viscosity for the RJ-based rejuvenator, while its ductility value is the largest. On the contrary, the softening point and viscosity of J3RAB rejuvenated bitumen are the highest, and the corresponding ductility value is the lowest. On the other hand, for the RX-based rejuvenator, the X2RAB binder with the highest viscosity shows the highest softening point and smallest ductility. In contrast, the X5RAB binder with the lowest viscosity discloses the lowest softening point and largest ductility. Therefore, to balance the high-and-low temperature properties and workability, both J4 and X3 are recommended as compound rejuvenators of artificial-aged bitumen. Furthermore, the rejuvenation efficiency of compound rejuvenators on the physical properties of rejuvenated binders strongly depends on the type of pure rejuvenator. Compared to the RJ-based rejuvenators, the rejuvenated binders with RX-based rejuvenators exhibit higher ductility and lower softening point and viscosity. As a result, the rejuvenated bitumen with RX-based rejuvenator presents a better low-temperature cracking resistance and workability.

#### 4.2.2. Viscous Properties

**Figure 6:**
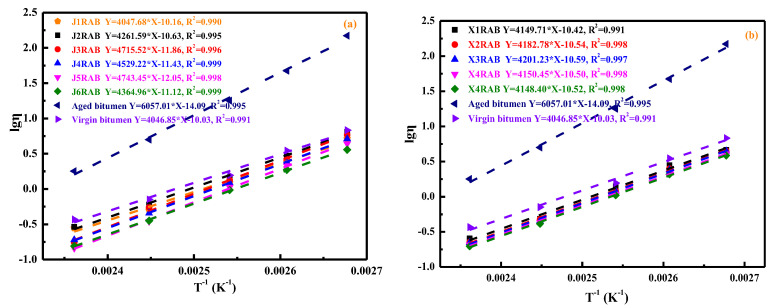
The correlation of viscosity and temperature for aged and rejuvenated bitumen.

The effects of compound rejuvenators on the viscosity and temperature sensitivity of rejuvenated bitumen were evaluated, and the results are displayed in Figure 6. As expected, with the temperature increases, the viscosity values of all samples decrease progressively, and there is a linearly-increasing relationship between the logarithm value of viscosity and the reciprocal of temperature. The aged bitumen has the most significant viscosity associated with the limited free volume and molecular mobility [18]. Therefore, incorporating a rejuvenator remarkably reduces the viscosity of artificial-aged bitumen. Interestingly, the viscosity values of all rejuvenated binders are lower than that of virgin bitumen. It implies that the rejuvenated binder with the same penetration grade as virgin bitumen has a lower viscosity and better workability [20].

The viscosity-temperature correlation equations of the virgin, aged, and rejuvenated binders are also shown in Figure 6, and the slope value represents the temperature sensitivity [22]. It is demonstrated that the aged bitumen exhibits the highest slope value, indicating that its temperature susceptibility is strongest. Meanwhile, virgin bitumen presents the lowest slope value and minimal temperature sensitivity. The slope values of rejuvenated binders are all close but larger than that of virgin bitumen, denoting that adding a compound rejuvenator could restore the workability and temperature sensitivity of aged bitumen to the virgin binder level simultaneously [18]. Furthermore, the compound rejuvenator type affects the viscosity and temperature sensitivity of rejuvenated binders, especially RJ-based rejuvenators. As the viscosity grade of the compound rejuvenator increases, the viscosity parameter of the corresponding rejuvenated binder decreases gradually.

As mentioned before, the increased temperature would accelerate the reduction of bitumen viscosity. From the viewpoint of the atomic level, molecular thermal movement is promoted as the temperature rises. It would result in an increment in the intermolecular distance and a decrease in molecular interaction [17]. This is why the viscosity of the whole bitumen system decreases at high temperatures. The Arrhenius equation was employed to estimate the viscosity-temperature correlation quantitively:

(4) $$\mathrm{lg}\left(\eta \left(T\right)\right)=\frac{E_{\eta }}{2.303\mathrm{RT}}+\mathrm{lg}\left(A\right)$$

where η(T) refers to the bitumen viscosity at temperature T, Pa·s; R is the gas constant, 8.314 J/(mol·K); Eη represents the viscous activation energy, kJ/mol; and A is the pre-reference parameter.

Table 8 lists the activation energy of virgin, aged, and rejuvenated binders. The aging process enlarges the activation energy from 77.49 to 115.98 kJ/mol. Meanwhile, involving a compound rejuvenator significantly decreases the activation energy of the aged binder to the virgin binder level. It is worth mentioning that most rejuvenated binders exhibit higher activation energy values than virgin bitumen. It implies that these compound rejuvenators could not restore the activation energy completely [18]. Additionally, compared to the RJ-based rejuvenators, the RX-based rejuvenators greatly renew the activation energy of aged bitumen to the virgin binder level. In summary, considering the reversible degree of activation energy, the RX-based compound rejuvenators are more appropriate than the RJ-based ones.

#### 4.2.3. Low-Temperature Properties

**Figure 7:**
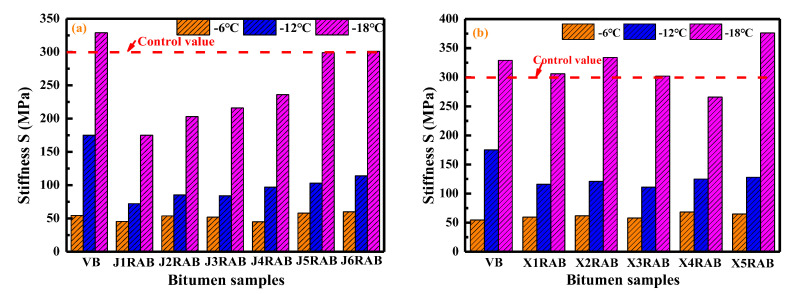
The stiffness values of rejuvenated bitumen at different temperatures.

In this study, the bending beam rheometer (BBR) tests were performed to assess the low-temperature cracking resistance of virgin bitumen and rejuvenated artificial-aged binders. Figure 7 illustrates the stiffness values of binders at different temperatures of −6, −12, and −18 °C, respectively. As expected, as the temperature descends, the S-values of all bitumen enlarge distinctly. The high bitumen stiffness at low temperatures is related to the reduction of free volume and enhancement of molecular interactions [18]. Regarding the RJ-based rejuvenated binders, as the viscosity grade of the compound rejuvenator rises, the S-value of the rejuvenated binder increases gradually. The RJ-based rejuvenated binder exhibits better low-temperature flexibility as the pure rejuvenator dosage increases. For RX-based rejuvenated binders, the difference in S-values is negligible, especially when the temperature is lower than −18 °C.

The stiffness values of rejuvenated binders are close or even lower than that of virgin bitumen, denoting that these compound rejuvenators effectively restore and improve the low-temperature cracking resistance of aged bitumen to the virgin binder level. Based on the previous study [34] and AASHTO T313 standard [35], the control S-value is 300 MPa, and the bitumen would suffer the cracking potential when the S-value exceeds the point. From Figure 7, the S-values of RJ-based rejuvenated binders at −18 °C are all lower than 300 MPa. It means that the RJ-based rejuvenators show a significant influence on weakening the low-temperature stiffness of aged bitumen. On the other hand, the RX-based rejuvenated binders behave with a larger S-value than the control value at −18 °C, except for the X4RAB binder. Overall, the restoration capacity of RJ-based rejuvenators on the low-temperature stiffness of aged bitumen is more significant than RX-based rejuvenators.

**Figure 8:**
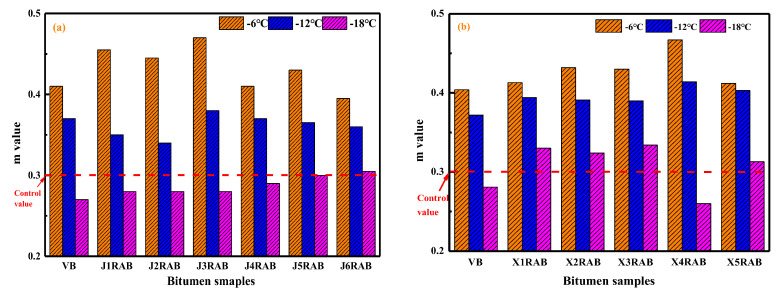
The m-value of rejuvenated bitumen at different temperatures.

The m-values of virgin and rejuvenated binders are displayed in Figure 8. It is demonstrated that the m-value of bitumen reduces as the temperature decreases. This is because the stiff bitumen molecules would need more time to accomplish the creeping stage with the external force at low temperatures [34]. Compared to a virgin binder, the m-values of rejuvenated binders are higher at all testing temperatures. The RJ- and RX-based compound rejuvenators efficiently retrieve and enhance the flexibility of aged binders better than virgin ones. Interestingly, the m-values of RX-based rejuvenated binders are higher than RJ-based rejuvenated bitumen. In other words, although the RJ-based rejuvenators remarkably reduce the stiffness value, their efficiency in enlarging the m-value and flexibility of rejuvenated binders is inferior to the RX-based rejuvenators.

Moreover, the m-values of virgin and rejuvenated binders are all larger than 0.3 at both −6 °C and −12 °C. In contrast, the m-value of virgin and all RJ-based rejuvenated bitumen are lower than the control point at −18 °C. However, the m-values of all RX-based rejuvenated binders at −18 °C meet the control requirement, except for the X4RAB binder. Therefore, according to the results of S and m-values of rejuvenated binders, the J4 and X3 are recommended as the compound rejuvenators of artificial-aged bitumen.

#### 4.2.4. Performance-Grade (PG)

**Figure 9:**
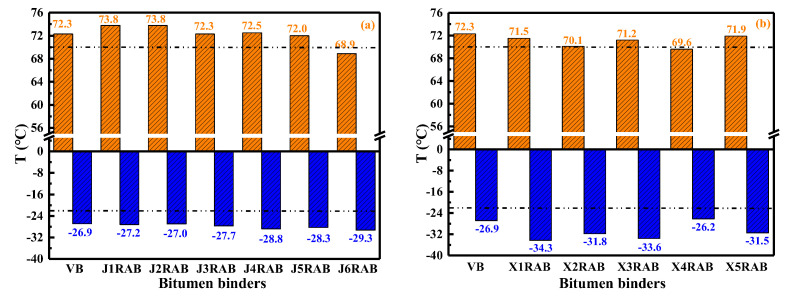
The Performance grade (PG) of rejuvenated binders.

The low-and-high temperature performance (PG) grades of virgin and rejuvenated binders are illustrated in Figure 9. The PG grades of all rejuvenated binders are close to that of virgin bitumen, indicating that the PG grade of aged binder could be restored to the virgin bitumen level using different types of RJ- and RX-based compound rejuvenators. However, the difference in the PG grade of rejuvenated binders is also observed, especially for the RX-based ones. Compared to the virgin bitumen, all rejuvenated binders display a lower low-temperature grade, especially for the X4RAB. Moreover, most RJ-based rejuvenated binders possess a larger high-temperature grade apart from the J5RAB and J6RAB binders. In comparison, all RX-based rejuvenated binders have a lower high-temperature grade than virgin bitumen. Herein, the RJ-based rejuvenated binders exhibit superior high-temperature grades, while the RX-based rejuvenators are more effective in strengthening the aged binder’s low-temperature grades. In conclusion, to ensure the high-and-low temperatures grade of the rejuvenated binder simultaneously, the compound rejuvenators J2, J4, X1, and X3 are recommended. The basis of recommending these optimum rejuvenators is from the balance of high-temperature and low-temperature performance grade of rejuvenated bitumen. It would be better for rejuvenated binders to exhibit a lower low-temperature PG value, but the compound rejuvenator cannot present a significant adverse effect on the high-temperature PG value of aged bitumen.

#### 4.2.5. Compatibility

It was reported that the difference between the measured and predicted viscosity values of rejuvenated bitumen was due to the incomplete compatibility between the rejuvenator and aged binder [16]. The difference parameter was utilized to estimate the compatibility of rejuvenated bitumen, and the predicted viscosity was calculated as follows:

(5) $$\mathrm{lg}\mu =x_{1}{\mathrm{lg}\mu }_{1}+x_{2}{\mathrm{lg}\mu }_{2}+x_{1}x_{2}G_{12}$$

where μ, μ_1_, and μ_2_ refer to the measured viscosity of rejuvenated bitumen, aged bitumen, and rejuvenator at one temperature, respectively; x_1_ and x_2_ are the mass fractions of aged bitumen and rejuvenator; G_12_ is the correction factor, which represents the difference between the measured and predicted viscosity of rejuvenated binder. The larger the G_12_ value is, the worse the compatibility between the rejuvenator and aged bitumen behaves [16].

Table 9 lists the 60 °C-viscosity and G_12_ parameter values of RJ-based rejuvenated binders. With the viscosity of a compound rejuvenator reduces, the absolute value of G_12_ parameter decreases dramatically. The J1RAB binder shows the highest absolute G_12_ value of 2.807, while the J6RAB binder has the lowest value of 0.417. The compound rejuvenator with a high dosage of pure rejuvenator and low viscosity exhibits better compatibility with artificial-aged bitumen. Moreover, Table 10 illustrates that the X1RAB and X5RAB show the largest and smallest G_12_ values, respectively. In addition, it is demonstrated that the RJ-based rejuvenators show lower G_12_ parameters and better compatibility with aged bitumen than RX-based rejuvenators.

#### 4.2.6. Aging Properties

In this study, the short- and long-term aging behaviors of rejuvenated binders were characterized using the penetration-based (AIP) and viscosity-based (AIV) aging index:

(6) $$\mathrm{AIP}=\frac{{\mathrm{Penetration}}_{\mathrm{aged}\;\mathrm{bitumen}}}{{\mathrm{Penetration}}_{\mathrm{unaged}\;\mathrm{bitumen}}}$$

(7) $$\mathrm{AIV}=\frac{{\mathrm{Viscosity}}_{\mathrm{aged}\;\mathrm{bitumen}}}{{\mathrm{Viscosity}}_{\mathrm{unaged}\;\mathrm{bitumen}}}$$

**Figure 10:**
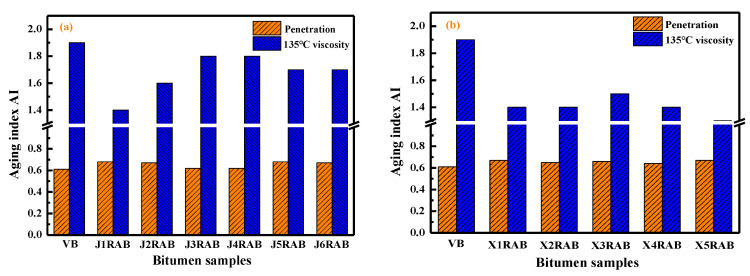
The aging index AI of virgin and rejuvenated binders after short-term aging.

**Figure 11:**
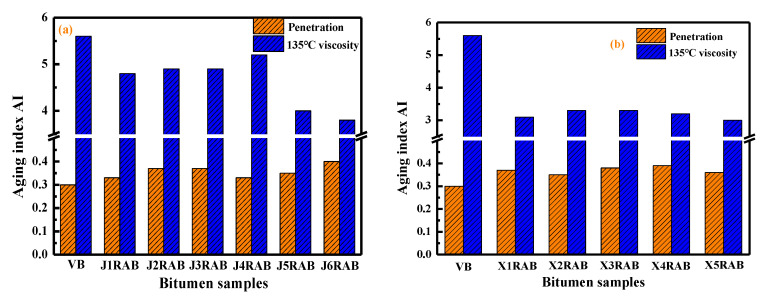
The aging index AI of virgin and rejuvenated binders after long-term aging.

Figure 10 and Figure 11 display the aging index of virgin and rejuvenated binders after the short-term and long-term aging process, respectively. The AIV values are larger than the AIP values of all bitumen binders, which is attributed to the definition difference between these two aging indexes. The bitumen with high AIP and low AVI values expresses a better aging resistance. As expected, with the increment in the aging degree, the AIP value of bitumen decreases while the AIV increases. All rejuvenated binders show higher AIP and lower AIV values than virgin bitumen. It implies that all rejuvenated binders exhibit a better anti-aging performance than virgin bitumen. The possible reason is that it is more difficult to oxidize lightweight rejuvenators and asphaltene than aromatic and resin molecules [40].

In addition, the aging properties of rejuvenated binders are strongly dependent on the rejuvenator type. The AIP and AIV values of RX-based rejuvenated binder are higher and lower than that of RJ-based rejuvenated bitumen regardless of the aging degree, respectively. It indicates that the RX-based rejuvenated binders exhibit a better aging resistance capacity than RJ-based ones. At the same time, the mass ratio of pure rejuvenator to virgin binder in compound rejuvenator also affects the aging properties of rejuvenated binders. The rejuvenated binder with J1 and J6 exhibits the best short-term and long-term aging resistance for RJ-based rejuvenators. Furthermore, the X5RAB binder presents the highest AIP and lowest AIV values after the short-and long-term aging processes. Therefore, it is summarized that the X5 compound rejuvenator is the best one to improve the aging properties of RX-based rejuvenated binders.

### 4.3. Rejuvenation of RAP-Aged Bitumen

#### 4.3.1. Physical Properties

After analyzing the rejuvenation efficiency of different compound rejuvenators on conventional, rheological, compatibility, and aging properties of artificial-aged bitumen, two types of rejuvenators (J4 and X3) are optimized to restore the properties of aged bitumen in reclaimed asphalt pavement (RAP) materials. As a result, the mass ratio of both rejuvenators to aged bitumen was the same as 43:57, and the related rejuvenated binders were marked as RB1 (J4) and RB2 (X3), respectively.

**Figure 12:**
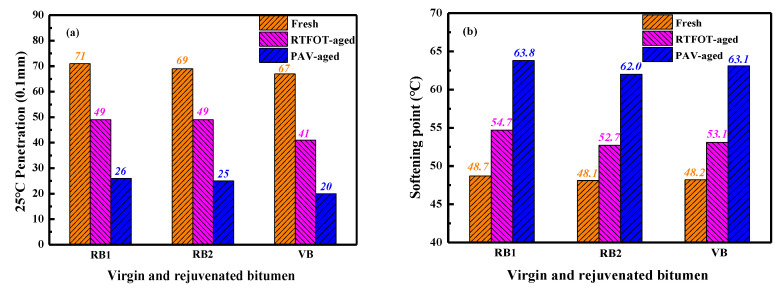

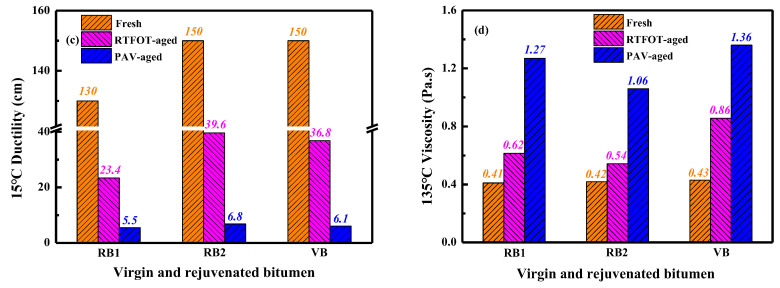
The physical properties of virgin and rejuvenated bitumen.

Figure 12 illustrates the physical properties of virgin and rejuvenated bitumen binders before and after RTFOT and PAV aging processes. These physical properties of two rejuvenated bitumen are both close to virgin bitumen. It indicates that the two rejuvenators are beneficial to efficiently rehabilitate the properties of RAP-aged bitumen to the virgin bitumen level. Moreover, the rejuvenator type dramatically influences the rejuvenation efficiency of conventional properties, especially ductility. Compared to the RB2 and virgin bitumen, the ductility value of RB1 is lower, implying that the rejuvenator J4 shows less rehabilitation effect on the low-temperature property than X3.

Meanwhile, the latter has a lower 135 °C-viscosity value than the former. Therefore, rejuvenator X3 exhibits better efficacy in enhancing the malleability and workability of the rejuvenated binder. Interestingly, the 135 °C-viscosity values of the two rejuvenated binders are smaller than virgin bitumen. The selected compound rejuvenators can reinstate the physical properties and workability of RAP-aged bitumen, which are even better than virgin bitumen.

In addition, the aging process decreases the penetration and ductility of virgin and rejuvenated binders, while the softening point and viscosity increase. As expected, the aging influence on conventional properties becomes more significant as the aging degree deepens. However, these physical parameters of rejuvenated binders are still close to virgin bitumen after short-term and long-term aging. Thus, it further verifies that the two compound rejuvenators can effectively regenerate the RAP-aged bitumen to the virgin binder level.

#### 4.3.2. Aging Resistance of Rejuvenated RAP Bitumen

To further evaluate the aging behaviors of rejuvenated binders, two parameters of residue penetration ratio (RPR) and viscosity ratio (VR) were calculated as follows:

(8) $$\mathrm{RPR}=\frac{{\mathrm{Pen}}_{\mathrm{aged}}}{{\mathrm{Pen}}_{\mathrm{unaged}}}*100\%$$

(9) $$\mathrm{VR}=\frac{\eta_{\mathrm{aged}}}{\eta_{\mathrm{unaged}}}$$

where the Pen_aged_ and Pen_unaged_ refer to the 25 °C-penetration value of bitumen before and after aging, while the η_aged_ and η_unaged_ are the 135 °C-viscosity value of aged and virgin binder, respectively.

**Figure 13:**
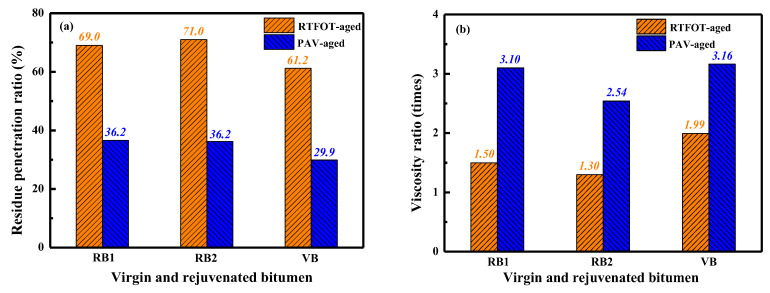
The aging properties of virgin and rejuvenated bitumen.

Figure 13 displays the residue penetration ratio and viscosity ratio of virgin and rejuvenated bitumen. The bitumen binder with higher RPR and lower VR value would present a greater aging resistance.

Compared to virgin bitumen, rejuvenated binders have a larger RPR and smaller VR value. In detail, after the short-term aging, the 25 °C-penetration value of RB1, RB2, and virgin bitumen decreases by 31%, 29%, and 38.8%, while the corresponding 135 °C-viscosity value increases by 50%, 30%, and 99%, respectively. Compared to the unaged samples, the penetration value of PAV-aged RB1, RB2, and VB binders drops by 63.8%, 63.8%, and 70.1%, while the viscosity rises by 210%, 154%, and 216%, respectively. Therefore, the aging resistance of rejuvenated binders is superior to virgin bitumen. At the same time, the RB1 binder exhibits a lower RPR and higher VR value than RB2, indicating that the rejuvenated bitumen with rejuvenator X3 shows better short- and long-term anti-aging performance than rejuvenator J4.

#### 4.3.3. Compatibility Parameters of Rejuvenated RAP Bitumen

To probe the compatibility between the RAP-aged bitumen and two compound rejuvenators, the correlation factor G_12_ is listed in Table 11. The parameter G_12_ refers to the difference between the theoretical and experimental viscosity values for rejuvenated bitumen. The compatibility between the aged bitumen and rejuvenator is better when the absolute value of the G_12_ parameter is lower. It is found that the G_12_ values of rejuvenated RAP bitumen are all close to the aforementioned rejuvenated binders J4RAB and X3RAB. It implies that the compatibility between the RAP-aged bitumen and two compound rejuvenators is sufficient. Furthermore, the absolute G_12_ value of the RB1 binder is lower than RB2, indicating that the compatibility between the RAP-aged bitumen and rejuvenator J4 is slightly superior to rejuvenator X3.

#### 4.3.4. Low-Temperature Properties of Rejuvenated RAP Bitumen

**Figure 14:**
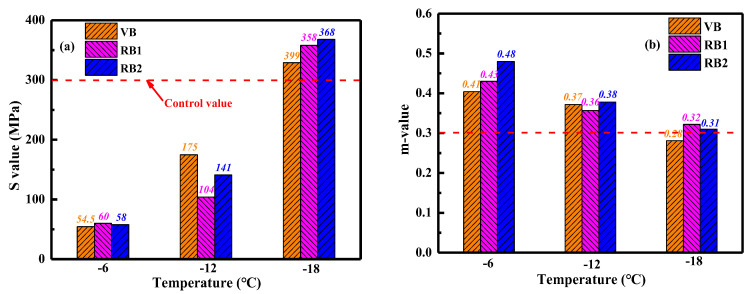
The s and m-value of virgin and rejuvenated bitumen.

The low-temperature properties of virgin and rejuvenated bitumen were characterized using the BBR tests at temperatures of −6, −12, and −18 °C. The stiffness (S) and m-value are illustrated in Figure 14. As expected, the stiffness values of all bitumen binders increase with the temperature decreases, while the m-values decline. It is attributed to the reduced free volume fraction and molecular mobility of bitumen molecules at low temperatures [38]. The difference in both S and m-values between the virgin and rejuvenated binders is insignificant, indicating that the two compound rejuvenators could restore the low-temperature cracking resistance of RAP-aged bitumen to the virgin binder level. According to the AASHTO T313 standard (s < 300 MPa & m > 0.3) [35], the virgin and rejuvenated binders meet the requirements when the testing temperature is not lower than –12 °C. The low-temperature performance-grade of virgin and rejuvenated bitumen attains the level of −22 °C.

#### 4.3.5. Rutting Resistance and Performance-Grade of Rejuvenated RAP Bitumen

The rutting factor G*/sinδ of the virgin, RAP-aged, and rejuvenated binders were measured, and there is a linearly decreasing trend of G*/sinδ value as the temperature increases. Because of the severe aging oxidation, the RAP-aged bitumen shows the maximum G*/sinδ value [39]. After adding the rejuvenators, the G*/sinδ value of RAP-aged bitumen distinctly declines, slightly lower than virgin bitumen. Although the compound rejuvenator enhances the low-temperature cracking and aging resistance properties of aged bitumen, it also adversely influences the high-temperature rutting resistance. Therefore, the rejuvenator dosage should be optimized to balance the high-and-low temperature properties of rejuvenated bitumen.

**Figure 15:**
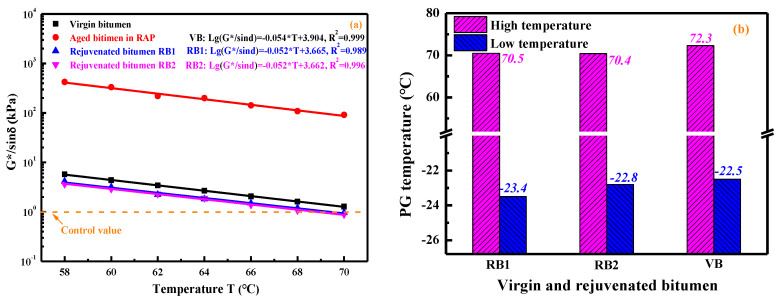
The rutting factor and PG grade of virgin and rejuvenated bitumen.

In addition, the failure temperature of bitumen was determined when the G*/sinδ value was equal to 1.0 kPa. Figure 15b shows the performance-grade of virgin and rejuvenated bitumen. It is demonstrated that the high-and-low temperature performance-grades of rejuvenated binders are close to virgin bitumen, although the latter slightly presents the higher PG values. It denotes that the two rejuvenators restore the performance-grade of RAP-aged bitumen to the virgin bitumen level. At the same time, the RJ-based rejuvenator exhibits superior low-temperature improvement capacity to the RX-based rejuvenator.

### 4.4. Recycling of Asphalt Mortar

In this study, the warm-mix recycled asphalt mortars were manufactured by mixing the rejuvenator X3 and oil-rich fine aggregate. The density and Marshall stability [37] of recycled mortars were measured to determine the warm-mix temperature and time. One group of recycled mortars was prepared at different warm-mix temperatures of 60, 90, 120, and 150 °C with a mixing time of 120 min to estimate the influence of warm-mix temperature. At the same time, another group of recycled mortars was obtained at 150 °C with a variable mixing time of 0, 60, 90, and 120 min to consider the effect of warm-mix time.

**Figure 16:**
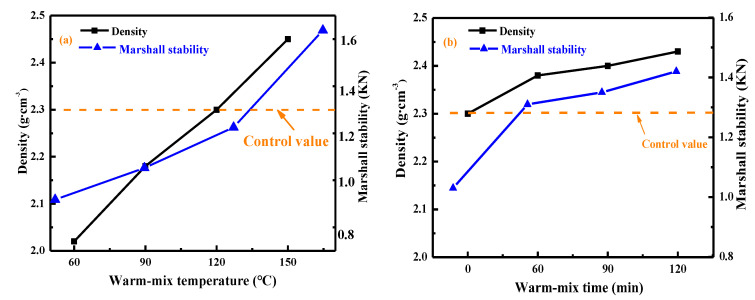
Influence of curing temperature and time on density and Marshall stability of recycled mortars.

The Marshall samples of recycled mortar with different curing temperatures and durations were prepared, and Figure 16 shows the results of Marshall stability and density. The Marshall stability values of recycled mortars are all lower than the corresponding asphalt mixture, which is attributed to the absence of coarse aggregates in mortar. Both the density and Marshall stability of recycled mortar rise as the increment in curing temperature and time. It implies that the curing temperature and time are beneficial to improving the adhesion properties of recycled mortar, which is associated with the larger diffusion and blending degree of rejuvenator into aged bitumen at higher mixing temperature and longer curing time.

When the warm-mix temperature rises from 60 to 150 °C, the density of recycled mortar increases from 2.02 to 2.45 g/cm^3^, while the Marshall stability enlarges from 0.99 to 1.62 kN. Meanwhile, when the warm-mix time extends from 0 to 120 min, recycled mortar’s density and Marshall stability increase by 5.7% and 37.9%, respectively. Hence, the influence of warm-mix temperature and time on the Marshall stability of recycled mortar is more apparent than the density parameter. Moreover, the warm-mix temperature influences recycled mortar more than the curing time. It is worth mentioning that the Marshall stability values of recycled mortars are all lower than the control sample. It demonstrates that the curing process could accelerate the blending of the rejuvenator with aged bitumen and restore the stickiness of aged bitumen [4]. Thereby, the adhesion and mechanical properties of asphalt mortars are improved gradually. However, due to the insufficient curing duration, the mechanical properties of recycled mortars are weaker than fresh mortar. On the basis of the physical and mechanical properties of recycled mortars, the temperature and time of 150 °C and 120 min are selected as the optimum curing conditions for following recycling of asphalt mixtures.

### 4.5. Recycling of Asphalt Mixtures

#### 4.5.1. Physical Properties of Recycled Asphalt Mixtures

**Figure 17:**
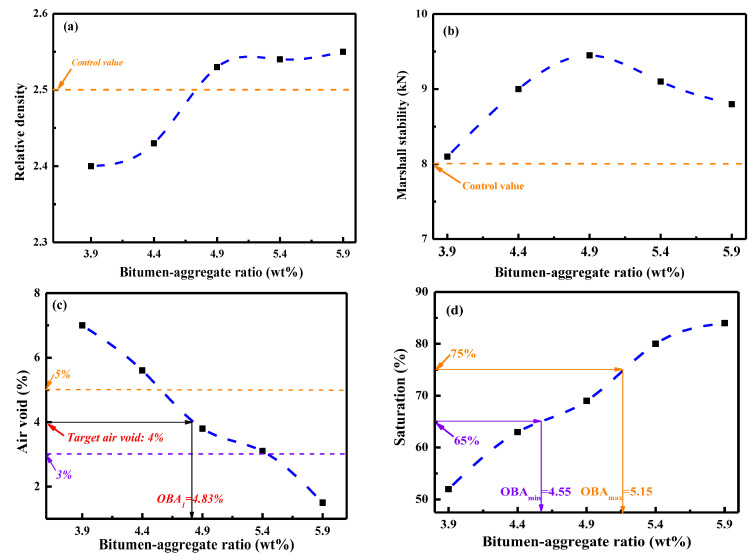
The influence of the bitumen–aggregate ratio on the basic properties of asphalt mixtures.

To determine the optimum bitumen–aggregate ratio of control and recycled asphalt mixtures, several groups of Marshall samples were fabricated with a variable bitumen–aggregate ratio of 3.9%, 4.4%, 4.9%, 5.4%, and 5.9%. Afterward, the relative density, air void, and saturation, Marshall stability values of these Marshall samples were measured [37], and the results are presented in Figure 17. It can be found that the bitumen–aggregate ratio greatly influences the volumetric and mechanical properties of recycled asphalt mixtures. As the bitumen–aggregate ratio increases, the relative density and saturation value rise distinctly, while the air–void parameter reduces. Meanwhile, as the bitumen–aggregate ratio increases, the Marshall stability value of recycled asphalt mixture enlarges first and then weakens, which reaches the maximum point when the bitumen–aggregate ratio is approximately 4.9%.

According to the technical specifications of the AC-13 asphalt mixture, the relative density and Marshall stability should be larger than 2.50 and 8 kN, respectively [37]. In addition, the corresponding air void and saturation should be in 3–5% and 65–75%. Therefore, in this study, the optimum bitumen–aggregate ratio of control and recycled asphalt mixtures was calculated using Equation (10):

(10) $$\mathrm{OBA}=\frac{{\mathrm{OBA}}_{1}+{\mathrm{OBA}}_{2}}{2}=\frac{{\mathrm{OBA}}_{1}+\frac{{\mathrm{OBA}}_{\min}+{\mathrm{OBA}}_{\max}}{2}}{2}$$

where the OBA refers to the optimum bitumen–aggregate ratio; OBA1 represents the bitumen–aggregate ratio when the air void is equal to the target value (4%); OBAmin and OBAmax is the minimum and maximum bitumen–aggregate ratio value when the saturation indicator reaches the minimum (65%) and maximum point (75%), respectively. These parameters are plotted in Figure 17, and the ultimate bitumen–aggregate ratio is determined as 4.8%.

#### 4.5.2. Rutting and Moisture Damage Evaluation of Recycled Asphalt Mixtures

**Figure 18:**
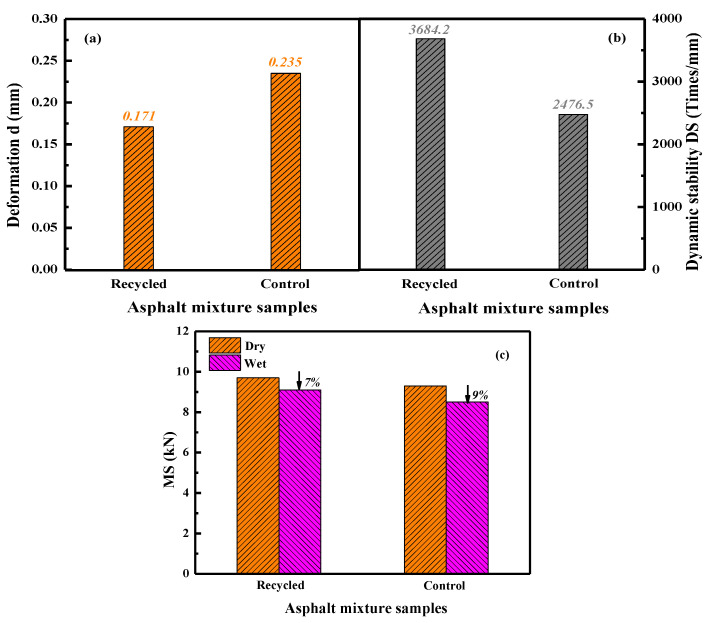
The rutting deformation (**a**), dynamic stability (**b**), and moisture sensitivity (**c**) of recycled and control asphalt mixtures.

The rutting and moisture damage resistance of recycled asphalt mixture is compared with the control sample to further validate the regeneration efficiency of the self-developed compound rejuvenator. Figure 18 illustrates the evaluation results of rutting and moisture damage for control and recycled asphalt mixtures. The asphalt mixture with higher dynamic stability and residue Marshall stability values would exhibit better rutting and moisture damage resistance [39]. It is illustrated that the dynamic stability values of control and recycled asphalt mixtures are both much larger than the standard requirement (>800 times/mm), indicating a sufficient rutting resistance capacity. Compared with the control sample, the deformation extent of the recycled asphalt mixture is lower, while its dynamic stability is about 1200 times/mm higher. It denotes that the rutting resistance of recycled asphalt mixture is superior to the fresh one. The aggregate gradation of the control and recycled asphalt mixture is the same. The greater rutting resistance of recycled asphalt mixture is mainly due to the predominant high-temperature properties of rejuvenated bitumen.

The Marshall stability and residue Marshall stability ratio of control and recycled asphalt mixture are presented in Figure 18c. The Marshall stability of recycled asphalt mixture is higher than the control sample, regardless of dry or wet conditions. Herein, the structural stability of recycled asphalt is better than the fresh one, which agrees well with the rutting evaluation results. As expected, the Marshall stability of the wet asphalt mixture is smaller than the dry sample, which is attributed to the adhesive performance loss because of moisture erosion [13]. However, the residue Marshall stability ratio of control and recycled asphalt mixture is higher than the standard requirement of 75%. It is worth noting that the residue Marshall stability ratio of the control sample is slightly lower than the recycled asphalt. Therefore, it can be summarized that the recycled asphalt mixture exhibits adequate structural stability, rutting, and moisture damage resistance performance.

## 5. Conclusions

This study comprehensively investigated the rejuvenation efficiency of various self-developed compound rejuvenators on the physical, rheological, and mechanical properties of artificial-aged and RAP-aged bitumen binders, asphalt mortars, as well as asphalt mixtures. Some main conclusions are listed as follows:

(1) The compound rejuvenators could rehabilitate the physical properties, workability, temperature sensitivity, and viscous behavior of artificial-aged bitumen to the virgin binder level. However, the ductility, viscosity, and workability parameters of various rejuvenated artificial-aged binders with the same penetration-grade were different. Based on high-and-low temperature properties and workability, the compound rejuvenators J4 and X3 were recommended to rejuvenate RAP-aged bitumen.
(2) The restoration capacity of RJ-based rejuvenator on the high-and-low temperature performance-grade of aged bitumen was more significant due to the better compatibility. Moreover, the RX-based rejuvenator was beneficial in enhancing the viscidity, flexibility, low-temperature grade, and aging resistance of the rejuvenated bitumen.
(3) Compared to compound rejuvenator J4, rejuvenator X3 exhibits better efficacy in improving the malleability, workability, and aging resistance of rejuvenated RAP-aged bitumen. In addition, the compatibility between the rejuvenator J4 and RAP aged bitumen was slightly superior to the rejuvenator X3.
(4) The density and Marshall stability of recycled asphalt mortar rise with the increment in curing temperature and time. Moreover, the temperature and time of 150 °C and 120 min were optimized as the curing conditions for recycling of asphalt mixtures.
(5) The relative density, saturation, and air-void parameters determined the optimum bitumen–aggregate ratio in recycled asphalt mixture as 4.8%. Moreover, the anti-rutting, structural stability, and moisture resistance of recycled asphalt mixtures were superior to the fresh ones, which further validated the rejuvenation efficiency of self-developed compound rejuvenators.

## Figures and Tables

**Table 1:** Physical properties and chemical components of virgin bitumen.

Properties	Measured Value	Test Standards
25 °C Penetration (0.1 mm)	67	ASTM D5 [26]
Softening point (°C)	48.2	ASTM D36 [27]
10 °C Ductility (cm)	84.8	ASTM D113 [28]
15 °C Ductility (cm)	>150	
Saturate (wt%)	13.33	ASTM D4124 [29]
Aromatic (wt%)	17.36	
Resin (wt%)	39.71	
Asphaltene (wt%)	29.60	

**Table 2:** Conventional properties of pure rejuvenators.

Properties		Rejuvenator J	Rejuvenator X
20 °C Density (kg·m^−3^)		0.985	0.997
60 °C Viscosity (Pa·s)		0.137	0.219
SARA analysis	Saturate S (wt/%)	73.24	56.84
	Aromatic A (wt/%)	22.85	36.13
	Resin R (wt/%)	3.64	6.96
	Asphaltene As (wt/%)	0.27	0.07
RTFOT aging	Mass change (wt/%)	−0.6	−0.4
	60 °C Viscosity-ratio	1.01	1.25

**Table 3:** Conventional properties of RAP binder and artificial-aged bitumen.

Properties	RAP Bitumen	Air-Blowing Aged Bitumen	Test Standard
25 °C Penetration (0.1 mm)	11	11	ASTM D5 [26]
Softening point (°C)	77.6	78.6	ASTM D36 [27]
15 °C Ductility (cm)	1.6	1.2	ASTM D113 [28]

**Table 4:** The technical requirements of rejuvenators.

Properties	R1	R5	R25	R75	R250	R500
60 °C viscosity (mPa·s)	50–175	176–900	901–4500	4501–12,500	12,501–37,500	37,501–60,000
TFOT viscosity ratio	≤3	≤3	≤3	≤3	≤3	≤3
TFOT mass change (wt%)	≤4, ≥−4	≤4, ≥−4	≤3, ≥−3	≤3, ≥−3	≤3, ≥−3	≤3, ≥−3

**Table 5:** The properties of different compound rejuvenators.

Rejuvenator Types	Viscosity Grade	Mass Percentage (wt%)		60 °C Viscosity (mPa·s)	TFOT Aging	
		AH-70	Rejuvenator		Mass Loss (wt%)	Viscosity Ratio
J1	RA-1	20	80	148	−2.1	1.05
J2	RA-5	40	60	505	−2.6	1.36
J3	RA-25	60	40	2570	−1.3	1.76
J4	RA-75	70	30	6213	−1.2	1.93
J5	RA-250	80	20	21,380	−0.7	2.05
J6	RA-500	87	13	52,710	−0.5	2.22
X1	RA-5	18	82	515	−0.86	1.33
X2	RA-25	47	53	2811	−0.54	1.35
X3	RA-75	60	40	6463	−0.14	1.62
X4	RA-250	80	20	24,950	−0.08	2.09
X5	RA-500	85	15	53,020	−0.19	2.28

**Table 6:** The aggregate gradation and binder dosage in RAP materials.

RAP Aggregates	Passing Percentage (wt%)										Binder Content
	16	13.2	9.5	4.75	2.36	1.18	0.6	0.3	0.13	0.075	
0–5mm	100	100	100	99.3	76	54	32.8	18.7	12.6	7.2	5.7%
5–10mm	100	100	93.5	39.5	22.5	16.1	10.6	6.3	4.1	2	3.7%
10–15mm	100	93	51	24.8	18.3	13.2	8.7	5.4	3.8	2.3	3.1%

**Table 7:** Physical properties of virgin and rejuvenated artificial-aged bitumen.

Bitumen Samples	J1RAB	J2RAB	J3RAB	J4RAB	J5RAB	J6RAB
Rejuvenator dosage (wt%)	24	27	36	43	54	68
25 °C penetration (0.1 mm)	73	63	65	66	69	70
Softening point (°C)	50.5	51.3	52.2	50.8	49.7	48.1
15 °C ductility (cm)	103.3	109.9	103.3	113	115.6	128.5
60 °C viscosity (Pa·s)	437.5	335.8	591.3	501.0	420.5	310.3
**Bitumen Samples**	**X1RAB**	**X2RAB**	**X3RAB**	**X4RAB**	**X5RAB**	**VB**
Rejuvenator dosage (wt%)	26	36	43	60	69	-
25 °C penetration (0.1 mm)	67	68	68	67	69	67
Softening point (°C)	48.5	50.3	49.3	48.6	47.7	48.2
15 °C ductility (cm)	109.8	108.2	126.6	150	150	150
60 °C viscosity (Pa·s)	378.3	397.5	343.1	298.1	276.5	307.0

**Table 8:** Viscous activation energy of aged and rejuvenated binders.

Samples	VB	J1RAB	J2RAB	J3RAB	J4RAB	J5RAB	J6RAB
Eη (kJ/mol)	77.49	77.50	81.59	90.29	86.72	90.83	83.58
**Samples**	**AB**	**X1RAB**	**X2RAB**	**X3RAB**	**X4RAB**	**X5RAB**	**-**
Eη (kJ/mol)	115.98	79.46	80.09	80.45	79.47	79.43	-

**Table 9:** G_12_ parameter of rejuvenated binders with RJ-based rejuvenators.

Rejuvenated Binders	Complex Rejuvenators			Aged Bitumen			Rejuvenated Binders		G_12_
	μ_1_ (Pa·s)	lgμ_1_	x_1_ (wt%)	μ_2_ (Pa·s)	lgμ_2_	x_2_ (wt%)	μ (Pa·s)	lgμ	
J1RAB	0.147	−0.83	24	25,800	4.41	76	437.5	2.64	−2.807
J2RAB	0.505	−0.29	27			63	335.8	2.52	−1.017
J3RAB	2.570	0.41	36			64	591.3	2.77	−0.865
J4RAB	6.213	0.79	43			57	501.0	2.69	−0.636
J5RAB	21.38	1.33	54			46	420.5	2.62	−0.498
J6RAB	52.71	1.72	68			32	310.3	2.49	−0.417

**Table 10:** G_12_ parameter of rejuvenated binders with RX-based rejuvenators.

Rejuvenated Binders	Complex Rejuvenator			Aged Bitumen			Rejuvenated Binders		G_12_
	μ_1_ (Pa·s)	lgμ_1_	x_1_ (wt%)	μ_2_ (Pa·s)	lgμ_2_	x_2_ (wt%)	μ_0_ (Pa·s)	lgμ_0_	
X1RAB	0.515	−0.28	26	25,800	4.41	74	397.5	2.59	−3.068
X2RAB	2.811	0.44	36			64	378.3	2.57	−1.767
X3RAB	6.463	0.81	43			57	343.1	2.54	−1.337
X4RAB	24.95	1.39	60			40	298.1	2.47	−0.536
X5RAB	53.02	1.72	69			31	276.5	2.44	−0.541

**Table 11:** Compatibility parameters of rejuvenated RAP bitumen.

	Rejuvenator			Aged Bitumen			Rejuvenated Bitumen		
	μ_1_/Pa·s	lgμ_1_	x_1_/%	μ_2_/Pa·s	lgμ_2_	x_2_/%	μ_0_/Pa·s	lgμ_0_	G_12_
RB1 (J4)	6.21	0.79	43	24,152	4.38	57	430.3	2.63	−0.84
RB2 (X3)	6.46	0.81	43			57	405.5	2.61	−0.97
